# Fusariosis in patients with hematological malignancies: Two case reports

**DOI:** 10.1016/j.mmcr.2023.08.005

**Published:** 2023-08-23

**Authors:** Jord W. Raymakers, Daan A.R. Castelijn, Caroline E. Rutten, Caspar J. Hodiamont

**Affiliations:** a Department of Medical Microbiology and Infection Control, Amsterdam University Medical Centers, Amsterdam, the Netherlands; b Department of Hematology, Amsterdam University Medical Centers, Amsterdam, the Netherlands

**Keywords:** Fusariosis, Fusarium species, Hematological malignancies, Cytarabine, Combination therapy

## Abstract

Immunosuppressed patients with hematological malignancies are at risk for invasive fungal infections (IFI), including infections with *Fusarium* species (spp.), which are increasingly reported. Particularly at risk are patients with acute myeloid leukemia (AML) treated with high-dose cytarabine as remission-induction therapy. Whether cytarabine increases the risk of IFI in comparison to other chemotherapy remains not fully determined. Additionally, no clear correlation between the in vitro established minimal inhibitory concentrations (MICs) of antifungal agents and clinical outcome has been established for fusariosis. To increase awareness and knowledge of invasive fusariosis, we report two cases of *Fusarium* spp. infections in neutropenic patients following treatment with cytarabine for AML. Despite high MICs for azoles both patients were treated with an azole in combination with liposomal amphotericin B. The combination therapy was successful in one patient, however the other patient did not survive the disseminated *Fusarium* infection.

## 1 Introduction

*Fusarium* species (spp.) are filamentous fungi that can be found in soil, plants and water [1]. More than 300 phylogenetically distinct species of *Fusarium* have been identified and over 70 species have been described as pathogenic in humans [2], mostly members of the complexes *F. solani* and *F. oxysporum* [3]. The main routes of infection are direct inoculation through traumatic injury or inhalation of airborne microconidia [3]. *Fusarium* spp. can cause keratitis or subcutaneous infection in immunocompetent patients [1]. Immunosuppressed patients with hematological malignancies are at risk for an invasive *Fusarium* infection [4]. Invasive fusariosis can manifest predominantly as fever, popular or nodular skin lesions with or without central necrosis, sinusitis, pneumonia, lymphangitis and/or cellulitis [1]. The diagnosis of fusariosis can be confirmed with growth of *Fusarium* spp. in culture of biological materials [1]. Unusual for invasive infections with filamentous fungi, up to 70% of the immunocompromised patients with invasive fusariosis have positive blood cultures [3]. A direct microscopic exam of skin lesions or respiratory materials can facilitate an early presumptive diagnosis when finding hyphae [1]. In addition, molecular diagnostic tests or mass spectrometry using matrix-assisted laser desorption–ionization flight time (MALDI-TOF) may be helpful [4]. A serum galactomannan test may be positive in invasive fusariosis in hematological patients, although considered specific for aspergillosis [1]. Where a decrease in C*andida albicans* infections has been documented after the introduction of fluconazole prophylaxis, an increase in invasive fungal infections (IFI), including fusariosis, has been described [5]. In high-risk hematologic patients with superficial skin lesions due to *Fusarium* spp. the occurrence of invasive fusariosis may be prevented by primary antifungal prophylaxis with azoles such as voriconazole or posaconazole [6]. Voriconazole and polyenes are recommended as primary treatment [3], although *Fusarium* strains with high minimum inhibitory concentrations (MICs) for these antifungal agents are frequently identified [1]. The 90 days survival after the diagnosis of invasive fusariosis is low, 60% and 53% when treated with voriconazole and amphotericin B, respectively. However this is still an improvement compared to the survival prior to the year 2000, possibly due to an increased awareness and changes in primary treatment [7].

The aim of this article is to increase the awareness and knowledge of fusariosis by presenting two cases of neutropenic patients following treatment with cytarabine for acute myeloid leukemia (AML). High-dose cytarabine is common practice in remission induction treatment of AML [8]. Although some have suggested that the risk for IFI is not increased with use of high-dose cytarabine compared to other chemotherapy, this has not yet been fully determined [9]. With the increase in reports and high mortality of this relatively rare mold, early recognition and prompt administration of adequate therapy regimes are of utmost importance [5,7].

## 2 Case presentation

**Fig. 1:**
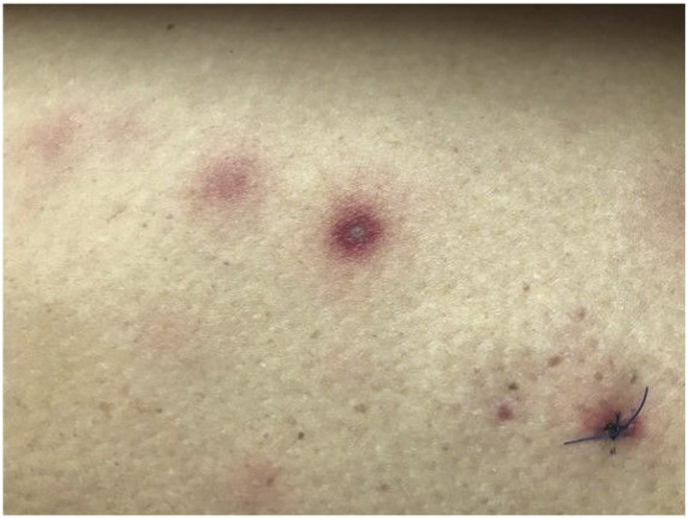
Picture of skin lesions.

A 39 year-old female, with a medical history of a metastatic Ewing sarcoma of the sacrum treated with chemotherapy and radiotherapy two years before, presented with a pancytopenia (week −11). The Ewing sarcoma was in stable partial remission and a biopsy of the bone marrow showed no signs of recurrence of the Ewing sarcoma. Following additional tests of the blood and bone marrow the patient was diagnosed with high risk myelodysplastic syndrome induced by chemotherapy and radiotherapy, which rapidly progressed to secondary AML. Since the patient reached the maximum cumulative dose of anthracyclines during treatment of the Ewing sarcoma the proposed treatment plan consisted of two induction remission courses of high dose cytarabine followed by an allogeneic hematopoietic stem cell transplantation (HSCT). At time of diagnosis there was profound neutropenia for which oral posaconazole prophylaxis was started (300 mg, once daily). On day 0 the patient was admitted to the hospital for remission induction chemotherapy with high dose cytarabine (1.5 g/m^2^ twice daily for 6 consecutive days). During admission in the hospital posaconazole blood levels were adequate (>0.8 mg/L). After five days (day +5) the patient had severe leukopenia (<0.1 × 10^9^/L). On day +23 the patient developed multiple painful erythematous lesions on body and face, some with dried-in vesicles (Fig. 1). A biopsy was taken from a lesion and sent to the pathology and microbiology departments for analyses. Polymerase chain reaction (PCR) tests for varicella zoster virus, herpes simplex virus and mycobacterium tuberculosis were negative. Bacterial culture showed no growth of pathogenic bacteria and no significant abnormalities were found on pathological examination at day +28. No signs of involvement of lungs or sinuses on CT-scan were found. In a new biopsy hyphae were found with direct microscopy. Upon recovery of neutrophils at day +31 the skin lesions slowly resolved and the patient was discharged from the hospital at day +33. A deeper biopsy of a skin lesion was taken for identification of the mold in advance of the second course of cytarabine. On day +52 a panfungal PCR identified the fungus as *Fusarium oxysporum* complex and shortly after the culture became positive. Meanwhile the patient was readmitted to the hospital. Considering the risk of further spread during a second neutropenic episode, another remission induction chemotherapy course was not considered feasible. Intravenous liposomal amphotericin B (3 mg/kg/day) was started and combined with oral voriconazole (200 mg twice daily after a loading dose). Susceptibility tests were performed and showed high MICs for posaconazole (>8 mg/L), itraconazole (>16 mg/L) and voriconazole (4 mg/L) and a low MIC for amphotericin B (0.5 mg/L). MICs were determined using the EUCAST reference method. After consultation of a Dutch mycology reference center combination therapy was continued despite high MICs for azoles for optimal treatment of the infection with *Fusarium* [3]. Later doses were readjusted according to the measured voriconazole blood levels. On day +76 the patient received a reduced intensity allogeneic HSCT. After two weeks of neutropenia the white blood count returned to normal without new manifestations of disseminated fusariosis and the liposomal amphotericin B was stopped shortly after. Three months later, after almost five months of voriconazole use, the voriconazole was stopped. No new signs of a relapse of fusariosis occurred afterwards.

A 56 year old female presented with a mild leukopenia and thrombocytopenia at a routine control (week – 6). Two years earlier the patient was treated for a good-risk AML with remission-induction chemotherapy followed by an autologous HSCT. In the Netherlands, autologous HSCT as consolidation for AML is considered for the following patients: <60 years old, diagnosed with intermediate or good-risk AML and measurable residual disease (MRD) negative after two remission-induction cycles [10,11]. After additional blood and bone marrow tests the patient was diagnosed with relapsed AML. The patient was admitted to the hospital on day 0 for re-induction chemotherapy with high-dose cytarabine (2 g/m^2^ twice daily for 6 consecutive days). If remission was achieved, consolidation with an allogeneic HSCT would follow. Yeast prophylaxis was started with fluconazole tablets (50 mg, once daily). According to local protocol there was no indication for prophylaxis against invasive mold infections as the patient had no expected long-lasting neutropenia or history of IFI. On day +7 the patient developed chemotherapy-induced neutropenia. Shortly after on day+12 a red, swollen and painful skin lesion of approximately 2 × 1 centimeters on the dorsal side of the third proximal interphalangeal joint of the left hand developed. Upon inquiry it appeared that while pruning roses one year ago the patient was pricked by a thorn in that specific finger. An X-ray of the finger did not show signs of a foreign body. Because arthritis was suspected, a joint puncture was performed. No crystals were detected by microscopy in the synovial fluid. However, not enough synovial fluid could be collected for culture. Empiric treatment for bacterial arthritis with flucloxacillin (6 g/day continuously) and gentamicin (7 mg/kg once) was started on day +14. The following days the lesion of the finger did not improve or progress, but the patient developed persistent neutropenic fever of unknown origin. Antibiotics were switched to vancomycin with ceftazidime.

**Fig. 2:**
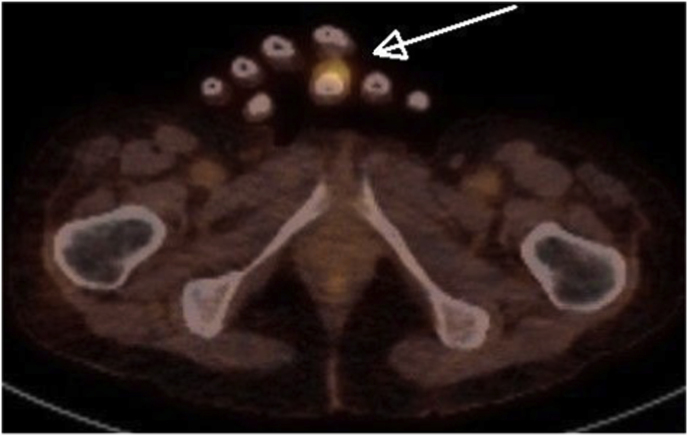
Axial plane of PET-CT with increased FDG uptake of the middle of the left hand.

**Fig. 3:**
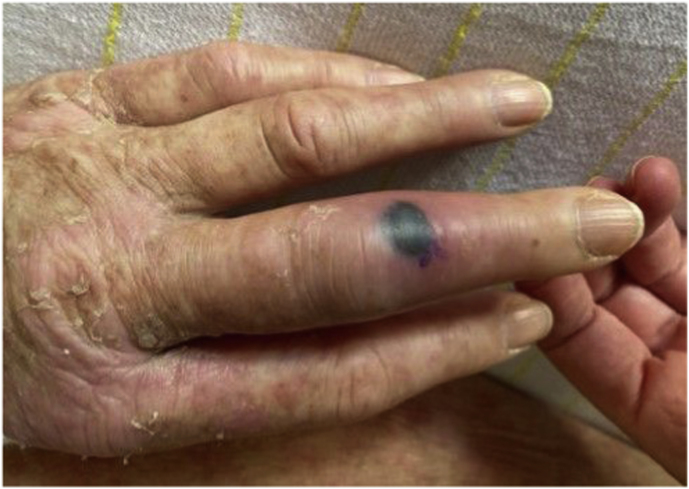
Picture of the necrotic lesion of the left middle finger.

**Fig. 4:**
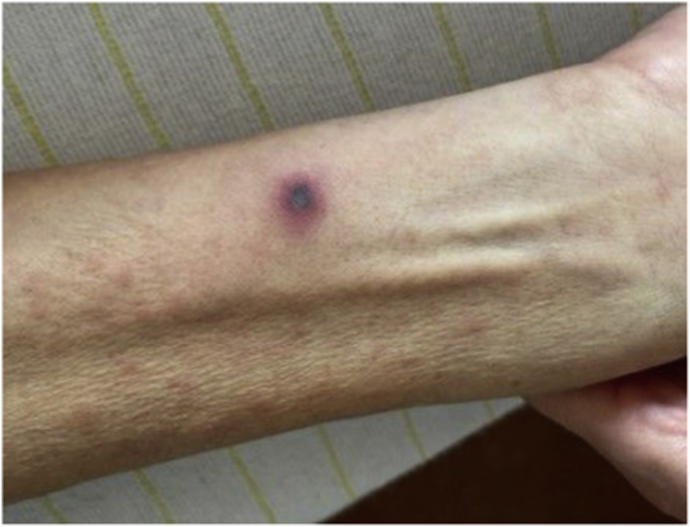
Picture of a necrotic lesion on the left lower arm.

**Fig. 5:**
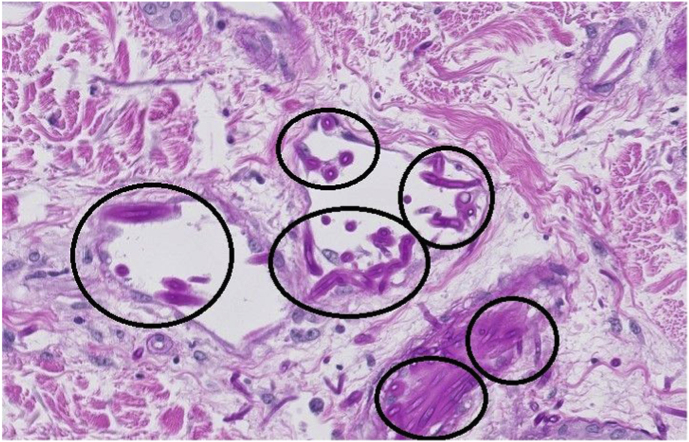
Image of a periodic acid-Schiff (PAS) stained histological skin biopsy with hyphae visible in black circles.

On day +20 a PET-CT showed a strongly increased fluorodeoxyglucose (FDG) uptake in the middle finger of the left hand (Fig. 2) and basal ganglia in the brain. No signs of involvement of lungs or sinuses on PET-CT were found. A small hyperechogenic fragment in the finger was found with high-resolution ultrasound suspect for a foreign body. The lesion on the finger evolved to a necrotic lesion (Fig. 3) and more necrotic skin lesions became visible on nose, arms and legs (Fig. 4). An ecthyma gangraenosum or IFI was suspected and anidulafungin with voriconazole was added to the antimicrobial therapy on day +23. Two biopsies of skin lesions were taken and showed hyphae with direct microscopy using periodic acid-Schiff (PAS) stain (Fig. 5). A switch in antifungal therapy was made to liposomal amphotericin B (5 mg/kg/day) in combination with voriconazole, since mucormycosis could not be excluded. The foreign body in the finger was surgically removed. The mucormycota species PCR and *Aspergillus* PCRs of the biopsy proved negative. On day +29 a blood culture taken during the use of anidulafungin and voriconazole became positive showing hyphae, highly suspicious for a *Fusarium* species. Voriconazole blood levels were consistently high (>4 mg/L) during treatment. The blood culture and the biopsy culture indeed showed growth of a *Fusarium* spp., later identified as *Fusarium solani* complex. Susceptibility testing using the EUCAST reference method showed high MICs for azoles (posaconazole >8 mg/L, isavuconazole >16 mg/L and voriconazole >16 mg/L) and anidulafungin (>16 mg/l) and an intermediate MIC for amphotericin B (2 mg/L). Additionally, olorofim was tested and showed a high MIC of >4 mg/L. The maximum tolerated dose liposomal amphotericin B (7.5 mg/kg/day) was started to optimize therapy and voriconazole was continued after consultation of a Dutch mycology reference center. As the risk was deemed low, and because of lack of therapeutic consequence due to the critical state of our patient, refractory AML was not ruled out. On day +37 the patients neutrophil count increased to 1.5 × 10^9^/L with support of granulocyte colony-stimulating factor (G-CSF) after 30 days of profound neutropenia. Clinically, new skin lesions and more intracranial lesions developed with neurological deterioration despite optimal therapy and a restored neutrophil count, suspect for progressive disseminated fusariosis. The clinical situation of the patient deteriorated and on day +42 the patient died.

## 3 Discussion

In this article we present two cases of invasive *Fusarium* infections in neutropenic patients that were treated for AML with high-dose cytarabine. By reporting these cases, we hope to increase awareness that this difficult-to-treat infection can occur in these patients. The role of high-dose cytarabine and the risk for invasive fungal infections in relation to other treatments is not yet fully determined [9]. Because of the scarcity of fusariosis, it will remain difficult to establish a relative risk ratio.

Where a plausible source of infection was found in case number two, namely a foreign body, no clear source of infection was found in case number one. *Fusarium* spp. usually enter the body through a pre-existing superficial lesion of the skin or nail or through inhalation, but no evidence was found for either route in case number one.

Primary antimold prophylaxis is usually indicated in hematologic patients who are at high risk for developing IFI, which is mainly intended to decrease the risk of invasive pulmonary aspergillosis [1]. Although the patient in case number one had received antimold prophylaxis for prolonged neutropenia, this did not prevented the development of skin lesions with *Fusarium* species. Breakthrough infections with *Fusarium* spp. in patients receiving antimold prophylaxis have been described frequently [1]. Possibly, if the patient in case number two had received antimold prophylaxis it might have prevented further spread to other organs, since the occurrence of widespread invasive fusariosis in high-risk hematologic patients with superficial skin lesions may be prevented by the use of primary antimold prophylaxis [6].

As primary treatment for fusariosis, the European Confederation of Medical Microbiology strongly recommends combination therapy with voriconazole and a lipid formulation of amphotericin B [3]. This because of the severity of the disease, difficulties in achieving voriconazole concentrations within target range and often high MICs for azoles and polyenes [3]. A step down to monotherapy may be possible if MICs are available [3]. However, no randomized controlled trials comparing monotherapy with combination therapy are known and overall response rates with monotherapy therapy were similar to combination therapy in retrospective studies [3]. In both cases presented in this article, the MICs of azoles were determined to be high, suggesting that azoles could be discontinued after susceptibility testing. However no clear correlation between MIC and outcome has been established for fusariosis and antifungal therapy [12,13]. EUCAST provides clinical breakpoints for yeasts and *Aspergillus* spp. but has not established breakpoints for *Fusarium* spp [14]. Therefore in vitro susceptibility testing may be helpful, but not indispensable in determining the choice of treatment of invasive fusariosis.

The optimal management of patients with fusariosis includes surgical debridement of infected tissues if possible, in addition to antifungal therapy [15]. In case number two the foreign body in the finger was surgically removed when hyphae were visible using direct microscopy on a biopsy. Further local debridement was not deemed meaningful as the skin lesions were widely spread.

Although both patients received combination antifungal therapy, the outcome differed between these patients. Even though the patients neutrophil counts recovered over time, the patient in case two died because of the severity and the advanced stage of invasive *fusariosis*. The 90-day probability of survival in patients with hematologic disease is less than 50% [7]. Persistent neutropenia and disseminated infection are associated with a poor outcome in patients with hematologic diseases and *Fusarium* infection [16]. A recovery of the host defenses is the most important prognostic factor [1,7], but in the patient in case number two recovery from neutropenia was not sufficient enough to control the already widely disseminated invasive *Fusarium* infection.

In conclusion, *Fusarium* infections in neutropenic patients with hematological malignancies are increasingly reported despite antimold prophylaxis for high-risk patients. Infections remain difficult to treat, with no clear correlation between MICs of antifungal drugs and patient mortality rates. Physicians should be aware of opportunistic infections with *Fusarium* spp. in neutropenic patients with hematological malignancies treated with high-dose cytarabine. Further studies are needed to establish optimal prophylaxis and treatment of these challenging infections.

## Declaration of competing interest

All authors declare no conflict of interest.
